# Infusion-Related Reactions Subsequent to Avelumab, Durvalumab, and Atezolizumab Administration: A Retrospective Observational Study

**DOI:** 10.3390/clinpract14020029

**Published:** 2024-02-23

**Authors:** Keiko Hata, Keina Nakamura, Shinichiro Maeda, Makiko Maeda, Yasushi Fujio, Sachiko Hirobe

**Affiliations:** 1 Laboratory of Clinical Pharmacology and Therapeutics, Graduate School of Pharmaceutical Sciences, Osaka University, Suita 565-0871, Japan; 2 Department of Pharmacy, Osaka University Hospital, Suita 565-0871, Japan; 3 Laboratory of Molecular Pharmaceutical Science, Graduate School of Medicine, Osaka University, Suita 565-0871, Japan; 4 Laboratory of Clinical Science and Biomedicine, Graduate School of Pharmaceutical Sciences, Osaka University, Suita 565-0871, Japan; 5 Integrated Frontier Research for Medical Science Division, Institute for Open and Transdisciplinary Research Initiatives, Osaka University, Suita 565-0871, Japan

**Keywords:** infusion-related reaction, immune checkpoint inhibitor, PD-L1, ADCC

## Abstract

Background: Avelumab, durvalumab, and atezolizumab are anti-programmed death-ligand 1 (PD-L1) antibodies approved for clinical application in Japan. Despite targeting the same molecule, avelumab elicits a different frequency of infusion-related reactions (IRRs) compared with durvalumab and atezolizumab, leading to differences in premedication recommendations. This study aimed to collect information to verify the relationship during IRRs and the characteristics of antibody molecules, by investigating the frequency of IRRs caused by three types of antibodies and the actual status of prophylactic measures. Methods: This single-center, retrospective observational study collected the medical records of 73 patients who received avelumab, durvalumab, or atezolizumab at Osaka University Hospital. Results: The frequency of IRRs was 50.0% (12/24) for avelumab, 31.0% (8/27) for durvalumab, and 18.2% (4/22) for atezolizumab. The IRRs were grade 2 in seven patients and grade 1 in five patients treated with avelumab, grade 2 in six patients and grade 1 in two patients treated with durvalumab, and grade 1 in all patients treated with atezolizumab. Among patients in whom symptoms were observed during the first administration, measures were taken to prevent IRRs for the second administration, but cases were confirmed in which symptoms reappeared, especially in patients who received durvalumab. Conclusion: Our findings indicate that the frequency of IRRs due to anti-PD-L1 antibodies is higher than that previously reported in clinical trials and different modifications in antibody molecules may affect the difference in IRR frequency.

## 1. Introduction

Many antibody drugs are being developed one after another for drug treatment of cancer. Among them, immune checkpoint inhibitors (ICIs) are therapeutic antibodies that counter the inhibition of T-cell activation [1]. In recent years, development has progressed, and the effects of not only ICI alone but also combination regimens with anticancer drugs have become clear, bringing about a major revolution in cancer treatment. ICI targets include programmed death receptor-1 (PD-1), anti-programmed death-ligand 1 (PD-L1), and cytotoxic T-lymphocyte antigen-4 (CTLA-4). In this study, we focused on antibodies targeting PD-L1, which have the same target, and several types have been approved in Japan, namely, avelumab (Ave), durvalumab (Dur), and atezolizumab (Atezo).

Infusion-related reactions (IRRs) are adverse reactions associated with antibody drugs that resemble hypersensitivity or allergic reactions. Symptoms of IRRs are nonspecific, and there are no clear diagnostic criteria. IRRs typically present as pyrexia, chills, rash, flushing, and itching and are believed to differ mechanistically from type I allergic responses [2,3,4,5]. It has been reported that the administration of rituximab, an anti-CD20 antibody, increases the concentration of cytokines such as TNF-α and IL-6 in the blood [6]. Though a previous study has suggested their mediation via cytokine syndrome, IRRs are more common at the first dose, when they show a greater response to premedication. Furthermore, the occurrence of IRRs may be linked to the proportion of antibodies derived from foreign proteins. There are four types of monoclonal antibodies, namely, mouse, chimeric, humanized, and human antibodies; the proportion of antibodies derived from foreign proteins increases in the order listed. If antibodies have many mouse-derived sequences, there is a risk to be recognized as a foreign substance when administered to a human. This may cause an allergic reaction or the production of antibodies against the administered antibody, weakening its effectiveness. As an example, rituximab is a chimeric antibody, the frequency of which has been reported to exceed 30% in non-Hodgkin’s lymphoma [7,8,9]. Conversely, although the anti-CD38 antibody daratumumab is a human antibody, it has strong antibody-dependent cellular cytotoxicity (ADCC) and induces the release of various cytokines, resulting in a high IRR frequency of 40–50% when administered intravenously [10]. Although there have been various discussions regarding the expression of IRRs, the mechanism of expression remains unclear, as most of the research reports on IRRs are concerned with their frequency and the risk factors for IRR expression.

Clinical trials have reported the frequency of IRRs as 12.0–21.5% for Ave (ClinicalTrials.gov number, NCT02155647, NCT02684006, and NCT02603432) [11,12,13], 1.7% for Dur (ClinicalTrials.gov number, NCT02125461) [14], and 1.4% for Atezo monotherapy (ClinicalTrials.gov number, NCT02409342) [15]. This difference may be accounted for by the attenuation of ADCC due to glycosylation modifications in the antibody’s Fc region. All antibodies are human immunoglobulin G1 (IgG1) antibodies. Ave and Dur are human antibodies, whereas Atezo is a humanized antibody. Human IgG1 antibodies induce ADCC via binding to Fcγ receptors (FcR) on natural killer cells and macrophages, which is attenuated by the substitution of interacting amino acids, especially in the case of FcγRIIIA. Though the nonglycosylated Ave induces ADCC against tumors [16], Dur and Atezo, which are glycosylated, attenuate ADCC.

Antihistamines, antipyretics, and analgesics are recommended premedications against IRRs caused by Ave. Premedications are recommended for use with Dur or Atezo in the second cycle for patients who experienced IRRs in their first cycle. Thus, disparities in the frequency of IRRs and usage of premedications exist despite targeting the same molecule.

The present study investigated the frequency of anti-PD-L1 antibody-mediated IRRs subsequent to Ave, Dur, and Atezo administration and the success of prophylactic measures for the same.

## 2. Materials and Methods

### 2.1. Patients

This single-center, retrospective observational study analyzed the data of 73 patients who received their first dose of Ave, Dur, or Atezo between August 2018 and October 2022 at Osaka University Hospital (OUH), Osaka, Japan. Data were collected from digital records. If IRRs occurred during the first administration, the IRR status was confirmed during the second and subsequent administrations. Patients who received concomitant intravenous administration of antineoplastic agents were excluded.

### 2.2. Assessments

Patient-related variables, including sociodemographic data, namely age, sex, prescribing department, height, weight, premedications, regimen, IRR development, and history of cardiac, pulmonary, or allergic disease; and blood laboratory findings, including white blood cell count (WBC), red blood cell count (RBC), hemoglobin, hematocrit, mean corpuscular volume (MCV), mean corpuscular hemoglobin (MCH), mean corpuscular hemoglobin concentration (MCHC), platelets, neutrophils, monocytes, lymphocytes, eosinophils, basophils, WBC count percentages, C-reactive protein (CRP), and the ratios of neutrophils, monocytes, and platelets to lymphocytes (NLR, MLR, and PLR, respectively); were analyzed. Laboratory tests were performed on the closest possible date prior to administering Ave, Dur, or Atezo. All blood tests were performed at the Laboratory for Clinical Investigation, OUH.

### 2.3. Anti-PD-L1 Antibody Administration

Ave was administered at a dose of 10 mg/kg at the Departments of Urology and Dermatology at OUH. Patients received 600 mg acetaminophen and 50 mg diphenhydramine hydrochloride as premedication on the same day. Additionally, axitinib was used along with other drugs at the Department of Urology. The first Ave dose was administered at 250 mL/h. Dur was administered at a dose of 10 mg/kg at the Department of Respiratory Medicine, with the initial dose at 100 mL/h. Atezo was administered at a dose of 1200 mg per cycle at the Department of Respiratory Medicine, with the initial dose at 270 mL/h. Premedication was not administered with the initial doses of Dur and Atezo.

### 2.4. IRRs

Patients’ symptoms were collected from the records of medical professionals such as doctors and nurses. IRRs were defined as adverse reactions that occurred within 24 h of drug administration and for which causal relationships could not be eliminated. IRR grades were determined as per the Common Terminology Criteria for Adverse Events, version 5.0 [17]. Grade 1 was defined as when some symptoms appeared, Grade 2 was defined as when some kind of response was taken to the symptoms that appeared, and Grade 3 was defined as when the responses to the symptoms that appeared were implemented multiple times.

Adverse reactions with symptoms that corresponded to basic words in the Medical Dictionary for Regulatory Activities (MedDRA, v26.0; www.meddra.org (6 February 2023), including pyrexia, chills, flushing, hypotension, dyspnea, wheezing, back pain, abdominal pain, and urticarial, were considered common infusion-related reactions (cIRRs) based on definitions used in the Ave clinical trial [13].

### 2.5. Prophylaxis

Prophylaxis (+) was defined as IRR prophylaxis at the second administration, including slower infusion rates than those in the aforementioned OUH regulations, and premedications, including antihistamines, antipyretics, and analgesics were according to practical guides for appropriate use in Japan.

### 2.6. Statical Methods

Categorical variables were compared using Fisher’s exact test, and continuous variables using the Wilcoxon signed-rank test. Statistical analyses were performed using JMP^®^ Pro (version 17.1.0; SAS Institute Inc., Cary, NC, USA), and statistical significance was set at *p* < 0.05.

## 3. Results

### 3.1. Comparison of IRRs at the First Infusion

**Figure 1:**
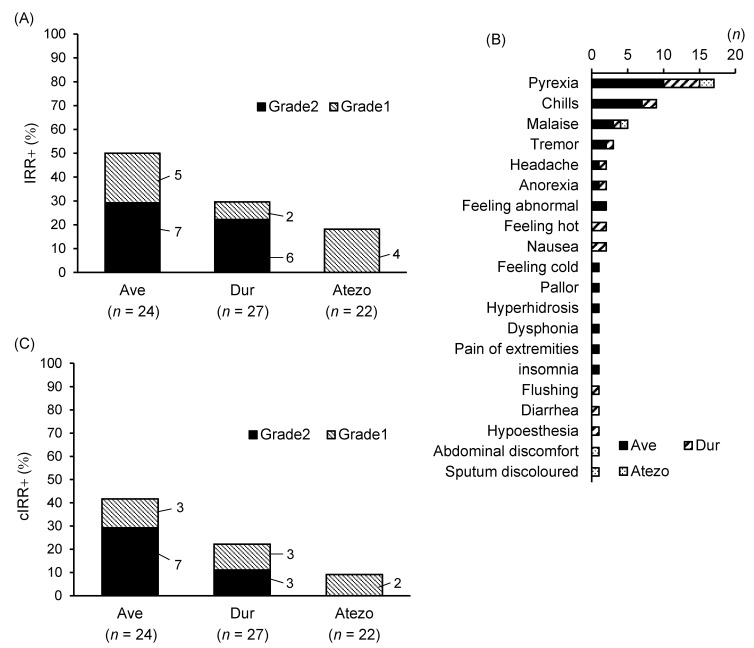
Frequency of infusion-related reactions (IRRs). (**A**) Number and percentages of IRR occurrence. (**B**) Symptoms observed. (**C**) Number and percentages of common IRR occurrence.

The frequency of IRRs was 50.0% (12/24) for Ave, 29.6% (8/27) for Dur, and 18.2% (4/22) for Atezo (Figure 1A). Among Ave-treated patients, seven had grade 2, and five had grade 1 IRRs. In Dur-treated patients, six had grade 2, and two had grade 1 IRRs. All patients had grade 1 IRRs in the Atezo-treated group. No cases of grade ≥ 3 IRRs were observed.

IRRs presented as pyrexia, chills, and fatigue (Figure 1B), with cIRRs being pyrexia and chills. The frequency of cIRRs was 41.7% (10/24), 22.2% (6/27), and 9.1% (2/22) for Ave, Dur, and Atezo, respectively (Figure 1C). Among Ave-treated patients, seven had grade 2, and three had grade 1 cIRRs. One individual who experienced grade 2 IRRs had grade 1 cIRRs, because of which a total of three patients had grade 2 cIRRs, and three had grade 1 cIRRs. Notably, all grade 2 IRRs were cIRRs.

### 3.2. Exploring Risk Factors for IRR Development

Patients were divided into IRR-presenting and nonpresenting groups to investigate the association between IRRs and patient background/clinical laboratory findings (Table 1). This revealed no contributory factors that caused the development of IRRs in Ave-, Dur-, and Atezo-treated patients. 

No significant associations between IRRs and clinical laboratory findings were observed in Ave- and Dur-treated patients. However, IRRs correlated significantly with higher WBC, neutrophil, and monocyte counts and lower MCHC, hemoglobin, and hematocrit in Atezo-treated patients (Table 2).

### 3.3. Comparison of IRRs and Prophylactic Measures at the Second Infusion

**Figure 2:**
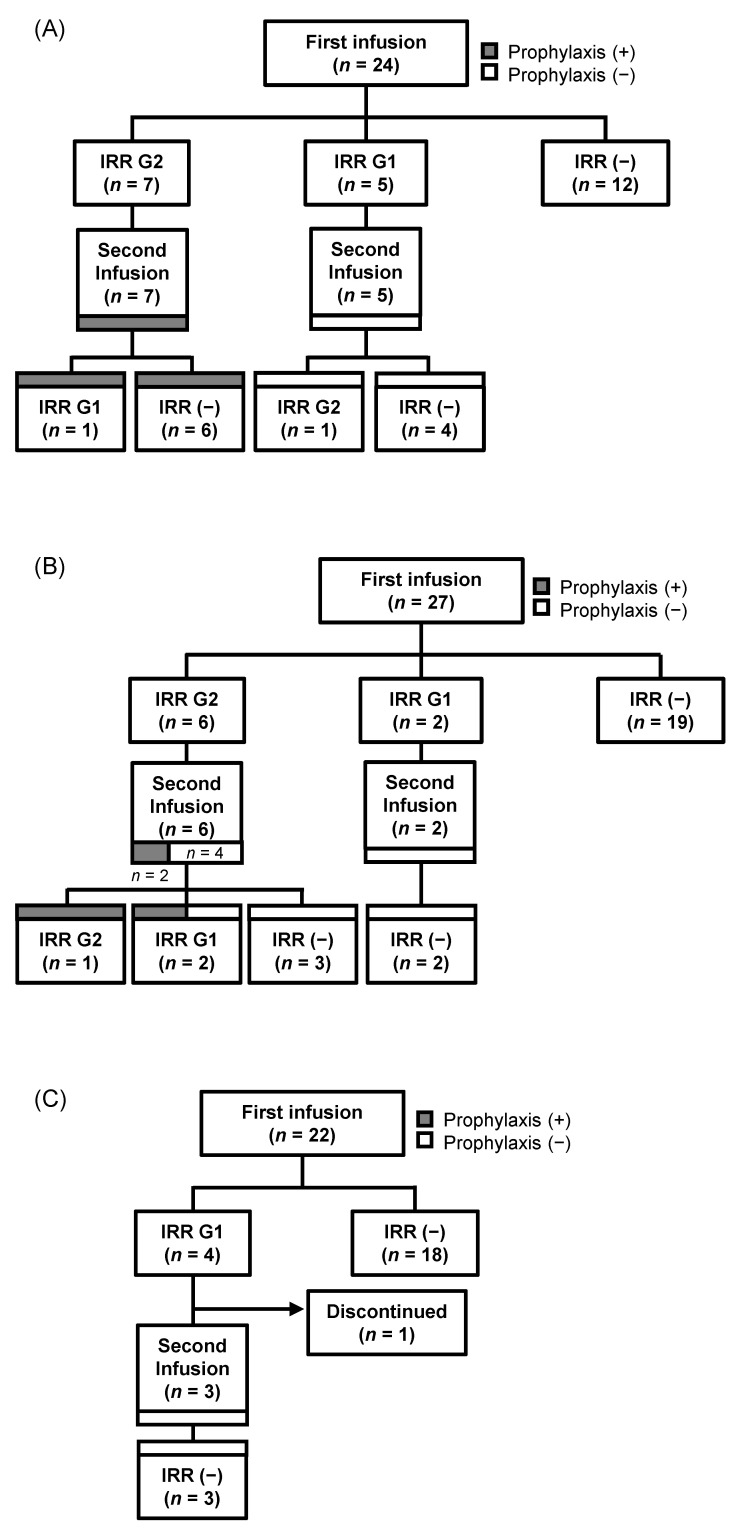
Flow diagram of the study results: (**A**) avelumab; (**B**) durvalumab; (**C**) atezolizumab.

The occurrence of IRRs was measured post-first and -second infusions of each drug, with prophylactic treatment before the latter was assessed (Figure 2A–C). Patients who developed grade 2 IRRs after the first Ave infusion were administered the second infusion at a slower rate after treatment with premedications, which reduced the subsequent frequency of IRRs (Figure 2A). Meanwhile, those who developed grade 1 IRRs were not administered prophylaxis before the second dose, of which one developed grade 2 IRRs. Of the six patients who experienced grade 2 IRRs after the first Dur infusion, two experienced IRRs after the second infusion, despite premedication in one case and a slower rate of administration in the other (Figure 2B). One patient who did not receive prophylaxis before the second infusion developed a grade 1 IRR. Further, two patients who developed grade 1 IRRs after the first Dur infusion did not receive prophylaxis before the second or develop IRRs. Of the four patients who developed grade 1 IRRs after the first Atezo infusion, three received the second. The second dose was not administered in the fourth case due to elevated tumor markers.

## 4. Discussion

The higher frequency of IRRs in our study, compared with that previously reported [11,12,13,14,15], might be accounted for by the inclusion of all adverse reactions for which causal relationships could not be eliminated. Therefore, we conducted an analysis of cIRRs based on symptoms that are considered to be characteristic of IRRs and defined in clinical trials of Ave [13]. Notably, the frequency of cIRRs was also high, suggesting not only that all symptoms were collected, but also, differences in the patient background from previous reports may have influenced the frequency of occurrence. However, the number of patients in this study was very small; hence, comparisons with reports from clinical trials were not appropriate. To implement safer treatments, it is necessary to confirm the frequency of IRRs in actual clinical settings by adding patients, conducting joint research with other institutions, and utilizing adverse event reporting databases. Additionally, several patients who developed IRRs after the first infusion did not develop IRRs after the second infusion, which corroborates previous observations of the occurrence of IRRs after the first infusion.

In this study, there were only three cases in which Ave was administered alone as a cancer treatment regimen; therefore, 21 patients who had combination therapy with axitinib were included in the analysis. The results of these 21 patients, excluding the three patients treated with Ave alone, confirmed that there were no major differences in the results of the 24 patients (Ave + axitinib and Ave alone). In cancer treatment regimens, the infusion of Ave and oral axitinib are started on the same day. Axitinib is a tyrosine kinase inhibitor of the vascular endothelial growth factor receptor. Although data on side effects that occur within 24 h of starting oral axitinib treatment are lacking, we cannot deny the possibility that side effects such as nausea, anorexia, and diarrhea may appear early during oral axitinib treatment. Furthermore, dysphonia is a characteristic side effect of drugs targeting vascular endothelial growth factor, and the IRRs of Ave are negative. We also evaluated the cIRRs by referring to the criteria defined in the clinical trial of Ave. Although the frequency of cIRRs decreased slightly, the frequency of IRRs remained high, and although the influence of axitinib could not be completely excluded, we believe that this information can be considered as an IRR due to Ave. However, further verification by increasing the number of patients treated with single-drug regimens and examining the effect of axitinib on IRR expression is warranted.

Though Dur and Atezo are reportedly associated with a low frequency of IRRs, we observed a higher frequency post the first dose. Additionally, few patients treated with Dur experienced IRRs after the second infusion despite prophylactics, with certain cases requiring continued hospitalization. Although premedications are used with Ave because of high IRR frequency, they are not the standard of care for Dur and Atezo monotherapy. This mandates further investigation of their role in the prevention of IRRs induced by the latter.

Dur and Atezo have reduced affinity for FcRIIIa and ADCC activity due to Fc region modifications. The three Dur heavy-chain constant region mutations, namely, L234F, L235E, and P331S, reduce binding to the complement protein C1q and affinity for FcγR [18,19]. Notably, although Dur’s affinity for FcR is unknown, antibodies with similar modifications demonstrate significant binding to FcR and ADCC activity in cell-based assays [20]. Further, Atezo lacks an N-linked sugar chain due to an asparagine to alanine substitution at position 298 in the CH2 domain of each heavy chain [21]. The distinctly modified Fc regions in Dur and Atezo may be responsible for observed differences in the frequency of IRRs in these therapies.

From the perspective of the proportion of foreign proteins, Atezo (humanized antibody) might have a higher frequency of IRRs than Dur (human antibody). However, this study showed that Dur-induced IRRs were more frequent, suggesting that the symptoms may be prolonged. This indicated that the frequency of IRRs may be influenced more by ADCC activity than by the proportion of foreign proteins. It is therefore important to verify the relationship between antibody characteristics and IRR occurrence.

The lack of studies on the risk factors for IRR development due to anti-PD-L1 antibodies highlights the need to develop methods for risk prediction and assessment of the actual development of IRRs in clinical settings.

The present study investigated IRR occurrence within clinical practice to bridge the gap in studies on IRRs caused by anti-PD-L1 antibodies. The limitations of this study included being a single-center, small sample-sized retrospective study, which might have led to target patient bias.

Furthermore, Ave administration differed from Dur and Atezo treatments, since premedications were offered only with the former. Though Ave had a greater frequency of IRRs, a lack of awareness on the part of healthcare professionals might have resulted in differential treatment of symptoms and subsequently skewed findings.

The present study expands the repertoire of known antibody drug characteristics, including IRR occurrence, antibody types, and glycosylation modifications, which will aid in the development of methods to predict the risk of and mitigate the frequency of IRRs after administering Dur and Atezo.

## Figures and Tables

**Table 1:** Comparison of patient characteristics with respect to frequency of IRRs.

Ave		All	Non–IRR	IRR	*p*-Value
		*n* = 24	*n* = 12	*n* = 12	
Renal cell carcinoma (Ave + axitinib)		21	10	11	
Bladder cancer (Ave)		2	1	1	
Merkel cell carcinoma (Ave)		1	1	0	
Age (years)		67.6 (33.8–86.1)	67.7 (37.8–86.1)	67.5 (33.8–86.0)	0.977 ^a^
	≥65 years (n [%])	16 (66.7)	7 (58.3)	9 (75.0)	0.667 ^b^
	<65 years (n [%])	8 (33.3)	5 (41.7)	3 (25.0)	
Body height (cm)		164.0 (143.4–184.1)	165.1 (143.4–184.1)	162.8 (145.0–178.3)	0.863 ^a^
Body weight (kg)		59.7 (39.8–113.9)	62.6 (41.2–113.9)	56.7 (39.8–74.0)	0.840 ^a^
Body mass index (kg/m^2^)		22.0 (15.8–36.3)	22.7 (15.8–36.3)	21.2 (16.8–24.5)	0.885 ^a^
Sex (n [%])	Male	15 (62.5)	8 (66.7)	7 (58.3)	1.000 ^b^
	Female	9 (37.5)	4 (33.3)	5 (41.7)	
Cardiovascular disease (n [%])	Yes	11 (45.8)	4 (33.3)	7 (58.3)	0.414 ^b^
	No	13 (54.2)	8 (66.7)	5 (41.7)	
Pulmonary disease (n [%])	Yes	8 (33.3)	5 (41.7)	3 (25.0)	0.667 ^b^
	No	16 (66.7)	7 (58.3)	9 (75.0)	
Drug allergy (n [%])	Yes	9 (37.5)	5 (41.7)	4 (33.3)	1.000 ^b^
	No	15 (62.5)	7 (58.3)	8 (66.7)	
Food allergy (n [%])	Yes	2 (8.3)	0	2 (16.7)	0.478 ^b^
	No	22 (91.7)	12 (100)	10 (83.3)	
Pollinosis (n [%])	Yes	5 (20.8)	1 (4.2)	4 (33.3)	0.317 ^b^
	No	19 (79.2)	11 (91.7)	8 (66.7)	
Asthma (n [%])	Yes	3 (12.5)	2 (16.7)	1 (8.3)	1.000 ^b^
	No	21 (87.5)	10 (83.3)	11 (91.7)	
**Dur**		**All**	**Non–IRR**	**IRR**	***p*-Value**
		***n* = 27**	***n* = 19**	***n* = 8**	
Age (years)		68.5 (44.7–82.0)	67.3 (44.7–80.0)	71.2 (56.8–82.0)	0.300 ^a^
	≥65 years (*n* [%])	19 (70.4)	13 (68.4)	6 (75.0)	1.000 ^b^
	<65 years (*n* [%])	8 (29.6)	6 (31.6)	2 (25.0)	
Body height (cm)		166.1 (146.7–180.3)	166.1 (156.5–179.4)	166.1 (146.7–180.3)	0.730 ^a^
Body weight (kg)		62.9 (39.9–100.1)	62.6 (42.7–95.5)	63.6 (39.9–100.1)	1.000 ^a^
Body mass index (kg/m^2^)		22.5 (15.7–30.8)	22.5 (15.7–29.7)	22.6 (18.1–30.8)	0.853 ^a^
Sex (n [%])	Male	21 (77.8)	15 (78.9)	6 (75.0)	1.000 ^b^
	Female	6 (22.2)	4 (21.1)	2 (25.0)	
Cardiovascular disease (n [%])	Yes	16 (59.3)	13 (68.4)	3 (37.5)	0.206 ^b^
	No	11 (40.7)	6 (31.6)	5 (62.5)	
Drug allergy (n [%])	Yes	8 (29.6)	5 (26.3)	3 (37.5)	0.658 ^b^
	No	19 (70.4)	14 (73.7)	5 (62.5)	
Food allergy (n [%])	Yes	2 (7.4)	2 (10.5)	0	1.000 ^b^
	No	25 (92.6)	17 (89.5)	8 (100)	
Pollinosis (n [%])	Yes	4 (14.8)	3 (15.8)	1 (12.5)	1.000 ^b^
	No	23 (85.2)	16 (84.2)	7 (87.5)	
Asthma (n [%])	Yes	1 (3.7)	1 (5.3)	0	1.000 ^b^
	No	26 (96.3)	18 (94.7)	8 (100)	
**Atezo**		**All**	**Non–IRR**	**IRR**	***p*-Value**
		***n* = 22**	***n* = 18**	***n* = 4**	
Age (years)		70.4 (42.6–82.9)	71.5 (59.2–82.9)	65.3 (42.6–75.0)	0.766 ^a^
	≥65 years (n [%])	18 (81.8)	15 (83.3)	3 (75.0)	1.000 ^b^
	<65 years (n [%])	4 (18.2)	3 (16.7)	1 (25.0)	
Body height (cm)		164.1 (148.6–181.5)	163.3 (148.6–174.6)	167.7 (155.6–181.5)	0.523 ^a^
Body weight (kg)		57.1 (36.7–70.6)	57.3 (36.7–70.6)	56.3 (47.4–61.6)	0.640 ^a^
Body mass index (kg/m^2^)		21.2 (15.4–27.9)	21.5 (15.4–27.9)	20.2 (16.9–24.7)	0.469 ^a^
Sex (n, %)	Male	15 (68.2)	12 (66.7)	3 (75.0)	1.000 ^b^
	Female	7 (31.8)	6 (33.3)	1 (25.0)	
Cardiovascular disease (n [%])	Yes	12 (54.5)	8 (44.4)	4 (100)	0.096 ^b^
	No	10 (45.5)	10 (55.6)	0	
Drug allergy (n [%])	Yes	7 (31.8)	5 (27.8)	2 (50.0)	0.565 ^b^
	No	15 (68.2)	13 (72.2)	2 (50.0)	
Food allergy (n [%])	Yes	6 (27.3)	5 (27.8)	1 (25.0)	1.000 ^b^
	No	16 (72.7)	13 (72.2)	3 (75.0)	
Asthma (n [%])	Yes	6 (27.3)	4 (22.2)	2 (50.0)	0.292 ^b^
	No	16 (72.7)	14 (77.8)	2 (50.0)	

Data were analyzed using (^a^) Wilcoxon rank-sum, (^b^) Fisher’s exact tests.

**Table 2:** Analysis of blood test parameters with respect to frequency of IRR.

Ave		All	Non–IRR	IRR	*p*-Value
		*n* =24	*n* =12	*n* =12	
WBC (10^3^/μL)		5.87 (3.46–8.35)	6.13 (3.67–8.35)	5.62 (3.46–8.16)	0.371 ^a^
RBC (10^6^/μL)		4.04 (2.68–5.84)	3.98 (3.39–5.01)	4.10 (2.68–5.84)	0.954 ^a^
MCV (fL)		92.5 (79.3–105.2)	92.9 (86.0–101.7)	92.1 (79.3–105.2)	0.665 ^a^
MCH (pg)		30.4 (24.5–33.6)	31.0 (27.3–32.8)	29.7 (24.5–33.6)	0.126 ^a^
MCHC (%)		32.8 (30.9–36.5)	33.4 (31.3–36.5)	32.3 (30.9–34.4)	0.078 ^a^
Hemoglobin (g/dL)		12.2 (8.2–15.9)	12.3 (10.3–14.7)	12.1 (8.2–15.9)	1.000 ^a^
Hematocrit (%)		37.1 (26.3–48.0)	36.8 (30.7–43.1)	37.4 (26.3–48.0)	0.665 ^a^
Platelets (10^3^/μL)		246 (160–430)	252 (189–373)	240 (160–430)	0.436 ^a^
Neutrophil	number (10^3^/μL)	3.84 (1.93–5.93)	4.12 (2.38–5.93)	3.56 (1.93–5.74)	0.248 ^a^
	ratio (%)	64.4 (45.5–72.4)	66.8 (51.5–71.8)	62.0 (45.5–72.4)	0.126 ^a^
Lymphocyte	number (10^3^/μL)	1.44 (0.81–2.55)	1.36 (0.81–2.16)	1.52 (1.08–2.55)	0.312 ^a^
	ratio (%)	25.4 (14.3–47.6)	22.5 (14.3–36.4)	28.3 (18.3–47.6)	0.112 ^a^
Monocyte	number (10^3^/μL)	0.38 (0.22–0.70)	0.40 (0.27–0.65)	0.36 (0.22–0.70)	0.194 ^a^
	ratio (%)	6.6 (3.3–11.2)	6.8 (3.3–9.7)	6.4 (4.6–11.2)	0.686 ^a^
Eosinophil	number (10^3^/μL)	0.17 (0.03–0.45)	0.21 (0.03–0.45)	0.14 (0.04–0.21)	0.751 ^a^
	ratio (%)	2.9 (0.7–6.0)	3.2 (0.7–6.0)	2.55 (0.9–4.5)	0.665 ^a^
Basophil	number (10^3^/μL)	0.04 (0.01–0.09)	0.05 (0.01–0.09)	0.04 (0.01–0.07)	0.371 ^a^
	ratio (%)	0.7 (0.2–1.6)	0.8 (0.2–1.5)	0.7 (0.2–1.6)	0.600 ^a^
NLR		2.9 (1.0–4.9)	3.3 (1.4–4.9)	2.4 (1.0–3.7)	0.100 ^a^
MLR		0.3 (0.1–0.7)	0.3 (0.1–0.7)	0.3 (0.1–0.6)	0.100 ^a^
PLR		181 (79–266)	197 (117–266)	164 (79–245)	0.157 ^a^
CRP (mg/dL)		1.26 (0.02–9.83)	1.21 (0.02–6.01)	1.31 (0.02–9.83)	0.397 ^a^
**Dur**		**All**	**Non–IRR**	**IRR**	***p*-Value**
		***n* = 27**	***n* = 19**	***n* = 8**	
WBC (10^3^/μL)		5.49 (1.25–19.32)	5.37 (1.25–19.32)	5.77 (3.78–9.24)	0.441 ^a^
RBC (10^6^/μL)		3.49 (2.27–4.36)	3.45 (2.27–4.27)	3.58 (2.81–4.36)	0.614 ^a^
MCV (fL)		95.1 (85.3–106.6)	95.0 (88.4–106.6)	95.3 (85.3–103.1)	0.577 ^a^
MCH (pg)		31.4 (27.4–35.4)	31.4 (27.6–35.4)	31.3 (27.4–32.8)	1.000 ^a^
MCHC (%)		33.0 (30.7–35.6)	33.1 (30.7–35.6)	32.8 (31.6–34.8)	0.472 ^a^
Hemoglobin (g/dL)		10.9 (6.8–14.3)	10.8 (6.8–12.7)	11.2 (8.7–14.3)	0.791 ^a^
Hematocrit (%)		33.1 (21.7–41.1)	32.7 (21.7–38.9)	34.0 (27.5–41.1)	0.790 ^a^
Platelets (10^3^/μL)		236 (97–358)	235 (118–358)	240 (97–357)	0.937 ^a^
Neutrophil	number (10^3^/μL)	3.45 (0.81–6.76)	3.31 (0.81–6.64)	3.81 (2.28–6.76)	0.690 ^a^
	ratio (%)	66.7 (20.5–86.4)	67.2 (20.5–86.4)	65.7 (52.4–79.0)	0.313 ^a^
Lymphocyte	number (10^3^/μL)	1.41 (0.21–14.57)	1.48 (0.21–14.57)	1.25 (0.47–3.47)	0.212 ^a^
	ratio (%)	20.8 (4.6–75.4)	20.2 (4.6–75.4)	22.2 (5.4–37.6)	0.203 ^a^
Monocyte	number (10^3^/μL)	0.47 (0.13–1.07)	0.44 (0.13–0.68)	0.53 (0.28–1.07)	0.614 ^a^
	ratio (%)	9.5 (2.8–14.8)	9.6 (2.8–14.8)	9.1 (6.7–12.4)	0.791 ^a^
Eosinophil	number (10^3^/μL)	0.13 (0.01–0.36)	0.12 (0.01–0.36)	0.14 (0.03–0.25)	0.381 ^a^
	ratio (%)	2.4 (0.1–6.5)	2.4 (0.1–6.5)	2.5 (0.7–4.6)	0.577 ^a^
Basophil	number (10^3^/μL)	0.03 (0–0.08)	0.03 (0–0.08)	0.04 (0.01–0.08)	0.652 ^a^
	ratio (%)	0.6 (0–1.5)	0.6 (0–1.5)	0.6 (0.2–1.0)	0.789 ^a^
NLR		5.1 (0.3–18.1)	5.3 (0.3–18.1)	4.7 (1.4–14.5)	0.193 ^a^
MLR		0.6 (0.0–2.3)	0.6 (0.0–1.8)	0.7 (0.2–2.3)	0.301 ^a^
PLR		327 (25–984)	351 (25–984)	268 (66–607)	0.232 ^a^
CRP (mg/dL)		1.68 (0.02–13.2)	1.63 (0.02–13.2)	1.79 (0.02–7.21)	0.595 ^a^
**Atezo**		**All**	**Non–IRR**	**IRR**	***p*-Value**
		***n* = 22**	***n* = 18**	***n* = 4**	
WBC (10^3^/μL)		7.90 (3.75–15.61)	7.16 (3.75–10.51)	11.22 (8.66–15.61)	0.024 *^,a^
RBC (10^6^/μL)		3.99 (2.51–5.63)	4.08 (2.75–5.63)	3.59 (2.51–4.85)	0.523 ^a^
MCV (fL)		92.8 (76.3–103.6)	94.1 (81.2–103.6)	87.1 (76.3–100.4)	0.328 ^a^
MCH (pg)		29.9 (22.7–33.7)	30.5 (26.8–33.7)	26.9 (22.7–31.5)	0.089 ^a^
MCHC (%)		32.2 (29.7–33.3)	32.5 (30.5–33.3)	30.8 (29.7–31.3)	0.008 *^,a^
Hemoglobin (g/dL)		11.9 (7.9–17.3)	12.4 (8.7–17.3)	9.3 (7.9–11.0)	0.019 *^,a^
Hematocrit (%)		36.8 (25.2–52.6)	38.3 (28.5–52.6)	30.2 (25.2–37.0)	0.045 *^,a^
Platelets (10^3^/μL)		299 (132–526)	283 (132–515)	372 (291–526)	0.055 ^a^
Neutrophil	number (10^3^/μL)	5.83 (2.36–14.03)	5.12 (2.36–8.15)	9.00 (7.07–14.03)	0.009 ^a^
	ratio (%)	71.8 (46.2–89.9)	70.1 (46.2–84.2)	79.5 (65.7–89.9)	0.136 ^a^
Lymphocyte	number (10^3^/μL)	1.39 (0.55–2.77)	1.40 (0.55–2.30)	1.37 (0.70–2.77)	0.580 ^a^
	ratio (%)	19.5 (4.5–45.1)	21.0 (9.2–45.1)	12.8 (4.5–24.2)	0.136 ^a^
Monocyte	number (10^3^/μL)	0.48 (0.11–0.93)	0.44 (0.11–0.93)	0.65 (0.43–0.75)	0.046 *^,a^
	ratio (%)	6.0 (2.8–9.9)	6.0 (2.8–9.9)	5.9 (4.7–7.6)	1.000 ^a^
Eosinophil	number (10^3^/μL)	0.16 (0–0.54)	0.17 (0–0.54)	0.15 (0.07–0.33)	0.832 ^a^
	ratio (%)	2.2 (0–7.8)	2.4 (0–7.8)	1.3 (0.6–2.9)	0.250 ^a^
Basophil	number (10^3^/μL)	0.04 (0.01–0.08)	0.03 (0.01–0.08)	0.05 (0.03–0.08)	0.136 ^a^
	ratio (%)	0.5 (0.1–1.5)	0.5 (0.1–1.5)	0.5 (0.2–0.7)	1.000 ^a^
NLR		5.3 (1.0–20.0)	4.4 (1.0–8.8)	9.4 (2.7–20.0)	0.136 ^a^
MLR		0.4 (0.1–1.1)	0.3 (0.1–0.8)	0.6 (0.3–1.1)	0.067 ^a^
PLR		259 (62–646)	235 (62–484)	368 (111–646)	0.251 ^a^
CRP (mg/dL)		1.43 (0.02–6.86)	1.08 (0.02–6.86)	3.01 (0.02–6.18)	0.268 ^a^

* Data were analyzed using (^a^) Wilcoxon rank–sum.

## Data Availability

Data were collected from the Hospital medical records. The data that support the findings of this study are available from the corresponding author upon reasonable request. Some data may not be made available because of privacy or ethical restrictions.

## References

[1] Chen D.S., Mellman I. (2013). Oncology meets immunology: The cancer-immunity cycle. Immunity.

[2] Peavy R.D., Metcalfe D.D. (2008). Understanding the mechanisms of anaphylaxis. Curr. Opin. Allergy Clin. Immunol..

[3] Gleich G.J., Leiferman K.M. (2009). Anaphylaxis: Implications of monoclonal antibody use in oncology. Oncology.

[4] Vultaggio A., Castells M.C. (2014). Hypersensitivity reactions to biologic agents. Immunol. Allergy Clin. N. Am..

[5] Lenz H.J. (2007). Management and preparedness for infusion and hypersensitivity reactions. Oncologist.

[6] Winkler U., Jensen M., Manzke O., Schulz H., Diehl V., Engert A. (1999). Cytokine-release syndrome in patients with B-cell chronic lymphocytic leukemia and high lymphocyte counts after treatment with an anti-CD20 monoclonal antibody (rituximab, IDEC-C2B8). Blood.

[7] Hayama T., Miura K., Uchiike A., Nakagawa M., Tsutsumi D., Sakagami M., Yoshida Y., Takei M. (2017). A clinical prediction model for infusion-related reactions to rituximab in patients with B cell lymphomas. Int. J. Clin. Pharm..

[8] Ohata S., Takenaka K., Sugiyama D., Sugimoto T. (2022). Bone Marrow Infiltration Is a Distinctive Risk Factor for Rituximab Infusion-Related Reactions in CD20-Positive B-Cell Non-Hodgkin Lymphoma. Adv. Hematol..

[9] Cho K.M., Keam B., Ha H., Kim M., Jung J.W., Song W.J., Kim T.M., Jeon Y.K., Kang H.R., Kim D.W. (2019). Clinical significance of rituximab infusion-related reaction in diffuse large B-cell lymphoma patients receiving R-CHOP. Korean J. Intern. Med..

[10] Palumbo A., Chanan-Khan A., Weisel K., Nooka A.K., Masszi T., Beksac M., Spicka I., Hungria V., Munder M., Mateos M.V. (2016). Daratumumab, Bortezomib, and Dexamethasone for Multiple Myeloma. N. Engl. J. Med..

[11] Kaufman H.L., Russell J., Hamid O., Bhatia S., Terheyden P., D’Angelo S.P., Shih K.C., Lebbe C., Linette G.P., Milella M. (2016). Avelumab in patients with chemotherapy-refractory metastatic Merkel cell carcinoma: A multicentre, single-group, open-label, phase 2 trial. Lancet Oncol..

[12] Motzer R.J., Penkov K., Haanen J., Rini B., Albiges L., Campbell M.T., Venugopal B., Kollmannsberger C., Negrier S., Uemura M. (2019). Avelumab plus Axitinib versus Sunitinib for Advanced Renal-Cell Carcinoma. N. Engl. J. Med..

[13] Powles T., Park S.H., Voog E., Caserta C., Valderrama B.P., Gurney H., Kalofonos H., Radulovic S., Demey W., Ullen A. (2020). Avelumab Maintenance Therapy for Advanced or Metastatic Urothelial Carcinoma. N. Engl. J. Med..

[14] Antonia S.J., Villegas A., Daniel D., Vicente D., Murakami S., Hui R., Yokoi T., Chiappori A., Lee K.H., de Wit M. (2017). Durvalumab after Chemoradiotherapy in Stage III Non-Small-Cell Lung Cancer. N. Engl. J. Med..

[15] Herbst R.S., Giaccone G., de Marinis F., Reinmuth N., Vergnenegre A., Barrios C.H., Morise M., Felip E., Andric Z., Geater S. (2020). Atezolizumab for First-Line Treatment of PD-L1-Selected Patients with NSCLC. N. Engl. J. Med..

[16] Boyerinas B., Jochems C., Fantini M., Heery C.R., Gulley J.L., Tsang K.Y., Schlom J. (2015). Antibody-Dependent Cellular Cytotoxicity Activity of a Novel Anti-PD-L1 Antibody Avelumab (MSB0010718C) on Human Tumor Cells. Cancer Immunol. Res..

[17] Common Terminology Criteria for Adverse Events (CTCAE). https://ctep.cancer.gov/protocoldevelopment/electronic_applications/ctc.htm#ctc_50.

[18] Oganesyan V., Gao C., Shirinian L., Wu H., Dall’Acqua W.F. (2008). Structural characterization of a human Fc fragment engineered for lack of effector functions. Acta Crystallogr. D Biol. Crystallogr..

[19] Stewart R., Morrow M., Hammond S.A., Mulgrew K., Marcus D., Poon E., Watkins A., Mullins S., Chodorge M., Andrews J. (2015). Identification and Characterization of MEDI4736, an Antagonistic Anti-PD-L1 Monoclonal Antibody. Cancer Immunol. Res..

[20] Wilkinson I., Anderson S., Fry J., Julien L.A., Neville D., Qureshi O., Watts G., Hale G. (2021). Fc-engineered antibodies with immune effector functions completely abolished. PLoS ONE.

[21] Herbst R.S., Soria J.C., Kowanetz M., Fine G.D., Hamid O., Gordon M.S., Sosman J.A., McDermott D.F., Powderly J.D., Gettinger S.N. (2014). Predictive correlates of response to the anti-PD-L1 antibody MPDL3280A in cancer patients. Nature.

